# Japanese and French translation and linguistic validation of a patient-reported outcome tool to assess quality of life in patients with Immune Thrombocytopenia (ITP): the ITP Life Quality Index (ILQI)

**DOI:** 10.1007/s12185-022-03382-0

**Published:** 2022-06-08

**Authors:** Yoshiaki Tomiyama, Stèphane Cheze, Laura Grant, Nicola Bonner, Sylvain Affinito, Mitsuhiro Nagano, Tanvi Rajput, Ricardo Viana

**Affiliations:** 1 Dept of Blood Transfusion, Osaka University Hospital, Osaka, Japan; 2 Institut d’Hematologie de Basse-Normandie, CHU Caen Hopital, Caen, France; 3 Adelphi Values Ltd, Adelphi Mill, Grimshaw Lane, Bollington, SK10 5JB Cheshire UK; 4 Novartis Gene Therapies, Zurich, Switzerland; 5 Novartis Pharma K.K., Tokyo, Japan; 6 Novartis Healthcare Pvt Ltd, Hyderabad, India; 7 Novartis Pharma AG, Basel, Switzerland

**Keywords:** Linguistic validation, Translation, Japanese, French, Immune Thrombocytopenia (ITP), Immune Thrombocytopenia Life Quality Index (ILQI)

## Abstract

**Objectives:**

The Immune Thrombocytopenia (ITP) Life Quality Index (ILQI) is a 10-item patient-reported outcome (PRO) measure developed in US-English to assess health-related quality of life (HRQoL) of adults with ITP. Analysis of ILQI responses indicated differences between Western and non-Western countries. The objective of this study was to translate and linguistically validate the ILQI for Japan and France.

**Methods:**

The ILQI underwent dual forwards/backwards translation with reconciliation and resolution. The translations were reviewed prior to conducting cognitive interviews with ITP patients (n = 5 Japan, n = 5 France). Analysis of interview transcripts highlighted required modifications to the ILQI translations. Japanese and French ITP experts reviewed the final translations for cultural relevance and appropriateness.

**Results:**

Most of the Japanese and French forward/backwards translations were reconciled with no revision. The ILQI instructions and items were well understood by Japanese and French participants. Wording in one item of the Japanese version of the ILQI was revised to better align with the source instrument. Three terms/phrases in the French translation were revised due to misunderstanding, being deemed inaccurate or culturally inappropriate. Following review by ITP experts from Japan and France, minor modifications were made.

**Conclusion:**

Findings confirm the linguistic validity of the ILQI in Japanese and French.

## Introduction

The Immune Thrombocytopenia (ITP) Life Quality Index (ILQI) is a 10-item patient-reported outcome (PRO) measure developed to assess health-related quality of life (HRQoL) of adult patients with ITP. The ILQI was developed to aid discussions between patients and clinicians and to inform treatment decisions. It is intended to be used as a tool in clinical practice to assess changes in HRQoL over time.

Clinical assessments of ITP severity often focus on platelet counts and risk of bleeding and do not always consider the patient’s quality of life [1]. Patients with ITP who do not bleed with low platelet counts, or who have sufficient counts to prevent frequent bleeding, may still experience significant impaired quality of life and have unmet needs [2]. The ILQI was developed to specifically target these patients and assess their quality of life.

The ILQI has relatively short instructions, which ask the patient to think about the ways ITP has affected their quality of life over the past month. All items employ a 4-point verbal rating scale (VRS) of ‘never, sometimes, more than half of the time and all of the time’. Items 1 and 2, which evaluate impacts on work/studying, include two additional response options to capture patients who are either ‘not working/studying due to ITP’ or ‘not working/studying due to other reasons’; item 5, which evaluates impacts on ‘sex life’ also includes a ‘not applicable/prefer not to say’ response option. The ILQI is a unidimensional scale, and a total score is calculated by adding each of the ten individual item scores. A minimum score of 7 is derived by patients answering at the lower end of the scale for every question in addition to also selecting ‘I am not working/studying due to other reasons’ for items 1 and 2 and answering ‘not applicable/prefer not to say’ to item 5 (‘sex life’). A maximum score of 40 is derived by patients answering at the higher end of the scale for every question. A higher score represents a greater impact on HRQoL. Cut points have been established whereby a total score of 17 suggests “impaired HRQoL” and a total score of 23–25 suggests “significantly impaired HRQoL”. A total score can be calculated with a maximum of three missing items.

**Fig. 1:**
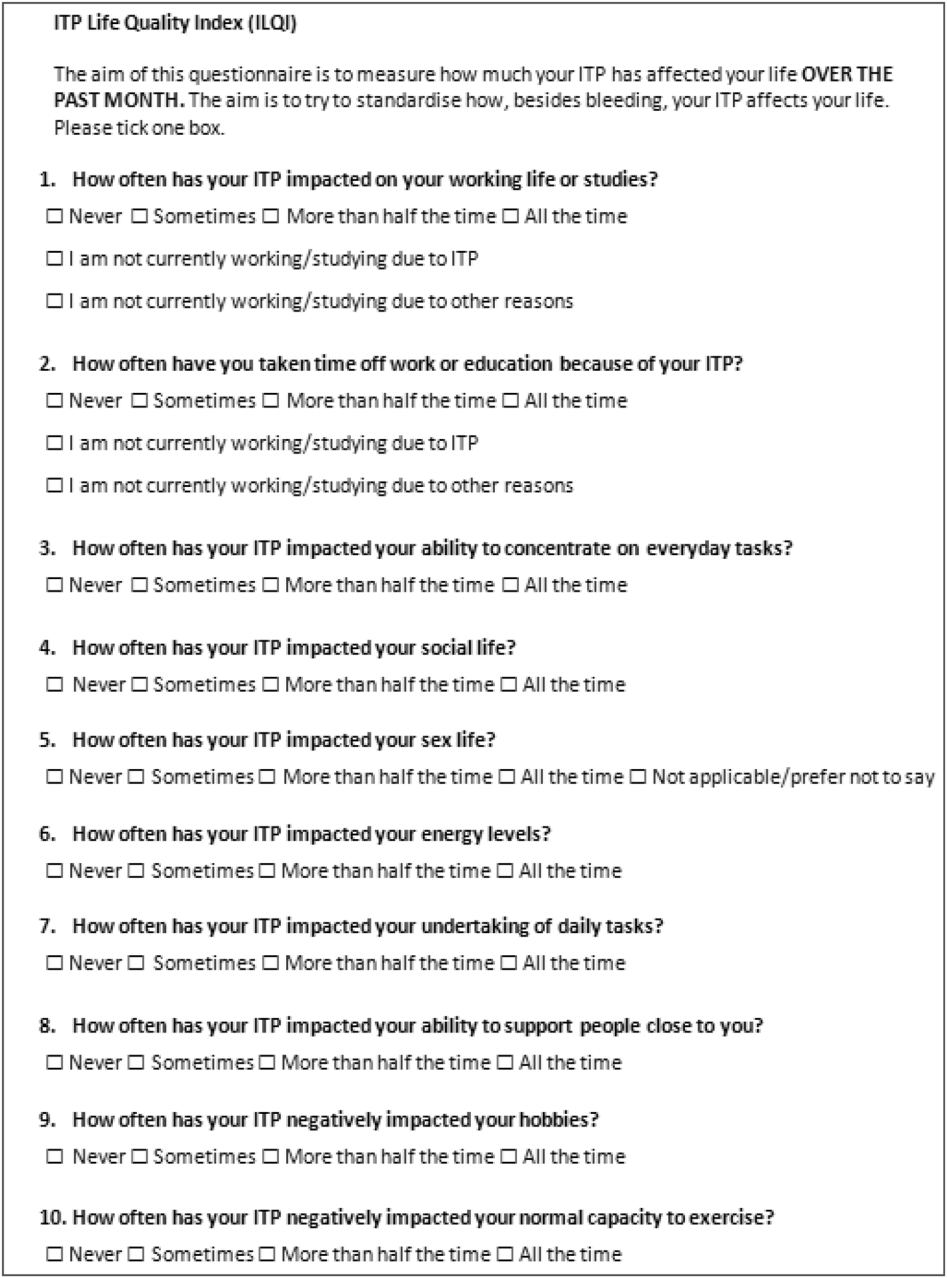
ILQI English version

The ILQI was originally developed in English by clinical experts in the field of ITP and content validity was confirmed by conducting individual qualitative interviews with 15 adult patients in the UK [3]. The ILQI was cognitively debriefed with ITP patients and items refined following qualitative analysis and additional clinical input. This qualitative work supported the content validity of the ILQI and confirmed that the concepts assessed are relevant and consistently understood and interpreted by adult patients with ITP [4]. The ILQI was then included in the ITP World Impact Survey (I-WISh), a global observational survey which collected data on the impact of ITP on 1507 patients’ HRQoL and collected data on physicians’ perceptions on using the ILQI in clinical practice [5, 6]. These findings confirmed the psychometric properties of the ILQI, specifically the validity and reliability of the ILQI to assess HRQoL, and confirmed the ILQI had good measurement properties [7]. The cut-off scores derived from the psychometric analysis helped to optimally discriminate between severity groups and aid patient-centered treatment decision making between patients and physicians. The English version of the ILQI is presented in Fig. 1.

The I-WISh included ITP patients from 13 different countries globally (USA, China, UK, France, Germany, Italy, India, Canada, Turkey, Japan, Colombia, Spain and Egypt), however, the aim of the survey was to primarily assess the psychometric properties of the ILQI and only one analysis was conducted to assess any differences between countries. Differential item functioning (DIF) was conducted to assess whether patients in one country answered each item in a similar way to the overall cohort of patients, stratified by disease severity [8, 9]. This analysis indicated large differences in the way patients from the USA and patients from non-Western countries (including Japan) answered most items in the ILQI.

From 2009 to 2011, ITP incidence rate in France was 2.9/100,000/year, with peaks among children and those over 60 years of age [10]. From 2004 to 2007, an overall incidence rate of ITP in Japan was 2.16/100,000/year, suggesting it is not markedly different from that of European countries [11]. The incidence rates highlight the need for a reliable and culturally appropriate assessment of HRQoL in ITP, for use in clinical practice in both Japan and France.

The primary aim of this study is to translate and linguistically validate the ILQI, using established methods, to maintain the validity when used in Japan. A secondary aim is to maintain the validity of the ILQI when used in France.

## Materials and methods

The ILQI was translated into Japanese for Japan and French for France following guidance from the World Health Organisation (WHO) and the International Society for Pharmacoeconomics and Outcomes Research (ISPOR) [12, 13]. Translation certificates are available to confirm the validity of each translation. The Japanese and French versions of the ILQI were also subject to linguistic validation analyses. The process workflow involved in the translation and linguistic validation is presented in Table 1.

**Table 1:** Process workflow involved in translation and linguistic validation

Process workflow	Method for the ILQI
Pre-flight definition of terms	The US-English version of the ILQI (i.e., source instrument) was reviewed and all concepts defined
Dual forward translation	Two separate native-speaking linguists from Japan and two native-speaking linguists from France were provided with the US-English version of the ILQI and independently performed forward translations, aiming for conceptual equivalence to the source instrument and cultural appropriateness for the target countries
Reconciliation	A third independent, native-speaking linguist from Japan and another from France compared the two forward translations, identifying any discrepancies or cultural differences to create a unified Japanese translation and a unified French translation
Back translation and resolution	A native-English speaking linguist with fluency in Japanese and another with fluency in French, translated the reconciled ILQI back into US-English using only the forward translation as source material
Resolution of Back and Forward Translation	A team consisting of native-speaking linguists fluent in Japanese or French and English resolved any discrepancies between the forward translation, back translation and original US-English version of the ILQI
Developer review	The reconciled forward and back translations of the Japanese and French ILQI were provided to the ILQI developers for review and comments were assessed by the linguistic teams from each country
Cognitive interviewing with patients	Qualitative interviews were conducted with five pre-screened respondents from Japan and five pre-screened respondents from France, who were representative of the ITP patient population, to test the translated versions of the ILQI. Of note, due to the rarity of ITP, the participants included in the cognitive interviewing were not diagnosed with ITP, but were diagnosed with a representative condition, for which the items on the ILQI were relevant. The aim of the interviews was to evaluate understanding of the translated ILQI. Updates were made to improve clarity and readability in the target language and to confirm consistency of understanding with the intended meaning
Review by ITP experts	The translated and linguistically validated Japanese and French versions of the ILQI were reviewed by one ITP expert from France and Japan respectively, to further assess cultural relevance. Experts were selected based on their ITP expertise and clinical experience and interest in PRO development

## Results

### Forward and backward translations and reconciliations

The detailed results from the forward and backwards translations and reconciliations for the translation of the ILQI into Japanese for Japan, and French for France, are presented in Tables 2 and 3, respectively. The ILQI was separated into the individual instructions, items, response options and scoring instructions. For each sentence/item, the key concepts were defined by providing a list of alternative words/terms with the same meaning, to aid interpretation and help to ensure an accurate translation. For example, the alternative words/terms for the concept of ‘aim’, which is included in the ILQI instruction, included ‘goal’, ‘intent’ and ‘purpose’. Each sentence/item then underwent forwards and backwards translation and reconciliation. Any revisions suggested by the developer or the linguists were noted and reconciled.

**Table 2:** Detail of forward and backwards translations and reconciliations for translation of the ILQI into Japanese for Japan

Source (US-English)	Concept	Reconciliation of Forward Translation (FT)	Back Translation (BT)	Resolution Reasoning	Developer Review	Linguist Feedback
TITLE OF ILQI ITP Life Quality Index (ILQI)	This is the title of the questionnaire The ITP Life Quality Index (ILQI), a 10-item patient-reported outcome (PRO) measure was developed as a tool for clinical practice to aid discussions between patients and physicians about disease experience so to inform patient-centric treatment decisions Using this tool, clinicians can better monitor symptoms beyond bleeding and rely on more than platelet counts when treating people with ITP "ITP" = immune thrombocytopenia	ITP生活の質指標 (ILQI)	ITP Quality of Life Indicator (ILQI)	No revision needed	No revision needed	No revision needed
FIRST SENTENCE OF ILQI INSTRUCTIONS The aim of this questionnaire is to measure how much your ITP has affected your life OVER THE PAST MONTH	"aim" = goal; intent; purpose "measure" = evaluate; assess "how much" = the extent to which; the degree to which "ITP" = immune thrombocytopenia "affected" = impacted "over the past month" = over the course of the last month; during the last month; in the previous month	この質問票の目的は、過去一ヵ月にわたり、免疫性血小板減少症 (ITP) が生活に与えた影響の度合いを測定することです。	The purpose of this questionnaire is to gauge the degree of impact immune thrombocytopenia (ITP) has had on your life over the past month	In the BT, "the degree" has been updated to "how much," which more accurately reflects the source. However, no revision is needed to the FT as the translation accurately conveys this meaning	No revision needed	No revision needed
SECOND SENTENCE OF ILQI INSTRUCTIONS The aim is to try to standardise how, besides bleeding, your ITP affects your life	"aim" = goal; intent; purpose "try to standardize" = make an effort to standardize; try to regulate "how" = the ways in which "ITP" = immune thrombocytopenia "affects" = impacts	また出血以外に、ITPがどのように生活に影響を及ぼすかを標準化する目的もあります。	Another purpose is to standardize how ITP impacts one’s life apart from bleeding	The FT has been revised to convey the source meaning of "the aim" and to remove the indication of "another." The BT has been revised to reflect the updated FT	No revision needed	No revision needed
THIRD SENTENCE OF ILQI INSTRUCTIONS Please tick one box	"tick" = check; place a tick mark or checkmark in	1つのボックスに印をつけてください。	Please check one box	No revision needed	No revision needed	No revision needed
ILQI ITEM 1 How often has your ITP impacted on your working life or studies?	"how often" = how frequently "ITP" = immune thrombocytopenia "impacted" = affected "your working life or studies" = your work life (refers to one's job, employment, ability to be present at work, complete one's work, etc.) "studies" = ability to attend classes, for example as well as accomplish what is needed/what he/she would like with regards to his/her studies	ITPによって、どのくらいの頻度で仕事や学業に影響がありましたか?	How often did ITP impact your [occupational] work and/or schoolwork?	The BT has been revised to remove bracket text and clarify the FT meaning of "studies" rather than "schoolwork." No revision is needed to the FT	No revision needed	No revision needed
ILQI RESPONSE OPTIONS □ Never □ Sometimes □ More than half the time □ All the time	"never" = not ever; at no point "sometimes" = from time to time; on occasion "more than half the time" = the majority of the time "all the time" = constantly; at all times	□ 全くない □ 時々 □ 過半数の時間 □ 常時	□ Never □ Sometimes □ Majority of time □ Always	No revision needed	Is there a closer translation for 'more than half of the time'? It feels like 'majority of the time' suggests almost all of the time, whereas 'more than half of the time' is a little lower than that	Concept states that "more than half the time" = the majority of the time", however, the FT and BT are revised to use a more literal translation
ILQI ADDITIONAL RESPONSE OPTION FOR ITEM 1 AND 2 □ I am not currently working/studying due to ITP	This is an available response choice "due to" = because of; as a result of "ITP" = immune thrombocytopenia	□ ITPのために現在、仕事や勉強はしていない	□ I am not working or studying now because of ITP	No revision needed	No revision needed	No revision needed
ILQI ADDITIONAL RESPONSE OPTION FOR ITEM 1 AND 2 □ I am not currently working/studying due to other reasons (0)	This is an available response choice "due to" = because of; as a result of "other reasons" = reasons other than ITP; other causes	□ 他の理由により現在、仕事や勉強はしていない (0)	□ I am not working or studying now because of other reasons (0)	No revision needed	No revision needed	No revision needed
ILQI ITEM 2 How often have you taken time off work or education because of your ITP?	"how often" = how frequently "taken time off work or education" indicates the person was absent from work or school; this could refer to unexpected absences or planned absences that are due to his/her ITP "ITP" = immune thrombocytopenia	のために、どのくらいの頻度で仕事を欠勤したり学校を欠席したりしましたか?	How often did you skip work or school because of ITP?	The BT has been revised to use "take time off" rather than "skip" for clarity of meaning. No revision is needed to the FT	No revision needed	No revision needed
ILQI ITEM 3 How often has your ITP impacted your ability to concentrate on everyday tasks?	"how often" = how frequently "ITP" = immune thrombocytopenia "impacted" = affected "to concentrate" = to focus your attention "everyday tasks" = common tasks; tasks that are typically part of everyday life "task" = piece of work to be done or undertaken; duty; chore	によって、どのくらいの頻度で日常作業を行う際の集中力に影響がありましたか	How often did ITP impact your concentration when you do your daily work/task?	The FT has been revised to reflect the source, perfect tense "has." The BT has been revised to reflect the updated FT and for consistent tense of "to do."	Could we remove the mention of work from this item to ensure it is still relevant for those patients not working	"Work" is removed from BT. No revision needed in FT
ILQI ITEM 4 How often has your ITP impacted your social life?	"how often" = how frequently "ITP" = immune thrombocytopenia "impacted" = affected "social life" = the domain of an individual's life that concerns engagement or interaction with other people	によって、どのくらいの頻度で社会生活に影響がありましたか?	How often did ITP impact your social life?	No revision needed	No revision needed	No revision needed
ILQI ITEM 5 How often has your ITP impacted your sex life?	"how often" = how frequently "ITP" = immune thrombocytopenia "impacted" = affected "sex life" = a colloquial term that captures all the aspects of one's life concerning sexual relationships and levels of sexual activity	によって、どのくらいの頻度で性生活に影響がありましたか?	How often did ITP impact your sex life?	No revision needed	No revision needed	No revision needed
ADDITIONAL RESPONSE OPTION FOR ILQI ITEM 5 □ Not applicable/prefer not to say	"never" = not ever; at no point "sometimes" = from time to time; on occasion "more than half the time" = the majority of the time "all the time" = constantly; at all times	□該当しない/回答したくない	□Not applicable/Don’t want to answer	No revision needed	No revision needed	No revision needed
ILQI ITEM 6 How often has your ITP impacted your energy levels?	"how often" = how frequently "ITP" = immune thrombocytopenia "impacted" = affected "energy" = the strength and vitality required for sustained physical or mental activity	によって、どのくらいの頻度で活力度に影響がありましたか?	How often did ITP impact your level of energy?	No revision needed	No revision needed	No revision needed
ILQI ITEM 7 How often has your ITP impacted your undertaking of daily tasks?	"how often" = how frequently "ITP" = immune thrombocytopenia "impacted" = affected "undertaking of" = taking part in; engagement in; management of "daily" = every day; on a daily basis "task" = piece of work to be done or undertaken; duty; chore	によって、どのくらいの頻度で日常作業の遂行に影響がありましたか?	How often did ITP impact the performance of your daily work/task?	Although the source uses the plural form "tasks," it has been confirmed that no revision is needed to the FT. The BT has been updated from singular "task" to plural "tasks."	Again, can we remove the mention of work to make sure it is relevant to all patients	"Work" is removed from BT. No revision needed in FT
ILQI ITEM 8 How often has your ITP impacted your ability to support people close to you?	"how often" = how frequently "ITP" = immune thrombocytopenia "impacted" = affected "to support people close to you" = to provide emotional support to people that are close to you (close friends, family, etc.)	によって、どのくらいの頻度で周りの人を手助けする能力に影響がありましたか?	How often did ITP impact your ability to help people around you?	The FT has been revised to better reflect the source "close" in order to convey emotional connection rather than physical location. The BT has been revised to reflect the updated FT and to use "support" rather than "help" to clarify the meaning conveyed in the source and FT	Could we remove the mention of 'emotional' as this item is meant to include any type of support (e.g. support with shopping, support with personal care and emotional support)	"Support" is deleted in both FT & BT
ILQI ITEM 9 How often has your ITP negatively impacted your hobbies?	"how often" = how frequently "ITP" = immune thrombocytopenia "impacted" = affected "hobbies" = activities done in one's leisure time for pleasure	によって、どのくらいの頻度であなたの趣味に悪影響がありましたか?	How often did ITP negatively impact your hobby?	Although the source uses the plural form "hobbies," it has been confirmed that no revision is needed to the FT. The BT has been updated from singular "hobby" to plural "hobbies."	No revision needed	No revision needed
ILQI ITEM 10 How often has your ITP negatively impacted your normal capacity to exercise?	"how often" = how frequently "ITP" = immune thrombocytopenia "impacted" = affected "normal capacity to exercise" = typical capacity to engage in physical activity to maintain and/or improve health	によって、どのぐらいの頻度で通常の運動能力に悪影響がありましたか?	How often did ITP negatively impact your normal motor ability?	The BT has been revised to more closely reflect the source "exercise." However, no revision is needed to the FT	No revision needed	No revision needed
INSTRUCTION AT THE END OF THE ILQI Please check you have answered EVERY question	"check" = make sure; be sure "answered" = responded to "every question" = all questions	すべての質問に回答したことを確認してください。	Please make sure that you have answered all questions	No revision needed	No revision needed	No revision needed
END OF THE ILQI Thank you	"thank you" is a closing message to the respondent indicating the end of the questionnaire	ありがとうございました	Thank you	Although missing in the source, ending punctuation has been added to the FT for correct grammar in the formatted file. The BT has been revised to reflect the updated FT	No revision needed	No revision needed
SCORING OF ILQI KEY:	The key below provides explanation of each score	解答:	Answer:	The FT has been revised to reflect the meaning of the source. The BT has been revised to reflect the updated FT	No revision needed	No revision needed
Never = 1, Sometimes = 2, More than half of the time = 3, All the time = 4,	"never" = not ever; at no point "sometimes" = from time to time; on occasion "more than half the time" = the majority of the time "all the time" = constantly; at all times "due to" = because of; as a result of "ITP" = immune thrombocytopenia	全くない = 1、時々 = 2、過半数の時間 = 3、 常時 = 4	Never = 1, Sometimes = 2, Majority of time = 3, Always = 4	No revision is needed		
I am not currently working/studying due to ITP = 4	This is an available response choice "due to" = because of; as a result of "ITP" = immune thrombocytopenia	ITPのために現在、仕事や勉強はしていない = 4	I am not working or studying now because of ITP = 4	No revision needed	No revision needed	No revision needed
Missing:		回答なし:	No answer:	No revision needed	No revision needed	No revision needed
I am not currently working due to other reasons = 0, not applicable/prefer not to say = 0	"due to" = because of; as a result of "other reasons" = reasons other than ITP; other causes	他の理由により現在、仕事や勉強はしていない = 0、該当しない/回答したくない = 0	I don’t work or study now because of other reasons = 0, Not applicable/Don’t want to answer = 0	The FT has been revised to remove "or study" in order to correctly reflect the source meaning. The BT has been revised to reflect the updated FT	No revision needed	No revision needed
Min score:		最低スコア:	Minimum score:	No revision needed	No revision needed	No revision needed
7		7	7	No revision needed	No revision needed	No revision needed
Max score:	"Min" = minimum	最大スコア	Maximum score:	No revision needed	No revision needed	No revision needed
40		40	40	No revision needed	No revision needed	No revision needed
Score of 20 or above suggests significantly impaired quality of life	"or above" = or higher "significantly" = sufficiently great or important to be worthy of attention; noteworthy "impaired" = negatively impacted; diminished "quality of life" = the standard of health, comfort, and happiness experienced by an individual or group	スコアが20点以上の場合は、生活の質に著しい障害が現れていることを示しています。	Score of 20 points or higher indicates the quality of life has experienced considerable [[or marked] impediments	The BT has been revised to more accurately reflect the FT and for consistency with the BT in the row below. No revision is needed to the FT	Is there an alternative translation for 'impediments'? Can you confirm that considerable and significant are a direct translation and are comparable?	"Impediments" is revised to "diminishment" in the FT and BT. "Considerable" is revised to "significant" in BT. No additional revision needed in FT
Score of 30 or above suggests severely impaired quality of life	"or above" = or higher "severely" = seriously "impaired" = negatively impacted; diminished "quality of life" = the standard of health, comfort, and happiness experienced by an individual or group	スコアが30点以上の場合は、生活の質に重度な障害が現れていることを示しています。	Score of 30 points or higher indicates the quality of life has experienced severe impediments	No revision is needed	Is there an alternative translation for 'impediments'?	"Impediments" is revised to "diminishment" in the FT and BT

**Table 3:** Detail of forward and backwards translations and reconciliations for translation of the ILQI into French for France

Source (US-English)	Concept	Reconciliation of Forward Translation (FT)	Back Translation (BT)	Resolution Reasoning	Developer Review	Linguist Feedback
TITLE OF ILQI ITP Life Quality Index (ILQI)	This is the title of the questionnaire The ITP Life Quality Index (ILQI), a 10-item patient-reported outcome (PRO) measure was developed as a tool for clinical practice to aid discussions between patients and physicians about disease experience so to inform patient-centric treatment decisions Using this tool, clinicians can better monitor symptoms beyond bleeding and rely on more than platelet counts when treating people with ITP "ITP" = immune thrombocytopenia	Indice de qualité de vie liée à la thrombopénie immune (ITP Life Quality Index, ILQI)	Immune thrombocytopenia-related life quality index (ITP Life Quality Index, ILQI)	No revision needed	No revision needed	No revision needed
FIRST SENTENCE OF ILQI INSTRUCTIONS The aim of this questionnaire is to measure how much your ITP has affected your life OVER THE PAST MONTH	"aim" = goal; intent; purpose "measure" = evaluate; assess "how much" = the extent to which; the degree to which "ITP" = immune thrombocytopenia "affected" = impacted "over the past month" = over the course of the last month; during the last month; in the previous month	L’objectif de ce questionnaire est de mesurer l’impact que la thrombopénie immune (TPI) a eu sur votre vie AU COURS DU DERNIER MOIS	The objective of this questionnaire is to measure the impact that immune thrombocytopenia (ITP) has had upon your life OVER THE PAST MONTH	Translations of "the goal" differs in the back translations (objective and goal used) it has been confirmed that no revision is needed as the translations are correct based on the context and meaning of the source	No revision needed	No revision needed
SECOND SENTENCE OF ILQI INSTRUCTIONS The aim is to try to standardise how, besides bleeding, your ITP affects your life	"aim" = goal; intent; purpose "try to standardize" = make an effort to standardize; try to regulate "how" = the ways in which "ITP" = immune thrombocytopenia "affects" = impacts	Le but est d’essayer de standardiser la manière dont votre TPI affecte votre vie, en dehors des saignements	The goal is to try to standardize how your ITP affects your life, apart from bleeds		Can we clarify that 'bleeds' is interpreted in the same way as 'bleeding'?	Confirming that in FR "saignements" can be translated as "bleeds" or "bleeding". The BT is revised for clarity with no revision to the FT needed
THIRD SENTENCE OF ILQI INSTRUCTIONS Please tick one box	"tick" = check; place a tick mark or checkmark in	Veuillez cocher une case	Please check off one box	No revision needed	No revision needed	No revision needed
ILQI ITEM 1 How often has your ITP impacted on your working life or studies?	"how often" = how frequently "ITP" = immune thrombocytopenia "impacted" = affected "your working life or studies" = your work life (refers to one's job, employment, ability to be present at work, complete one's work, etc.) "studies" = ability to attend classes, for example as well as accomplish what is needed/what he/she would like with regards to his/her studies	À quelle fréquence votre TPI a-t-elle eu un impact sur votre vie professionnelle ou vos études ?	How frequently has your ITP had an impact upon your professional life or your studies?	No revision needed	No revision needed	No revision needed
ILQI RESPONSE OPTIONS □Never □Sometimes □ More than half the time □ All the time	"never" = not ever; at no point "sometimes" = from time to time; on occasion "more than half the time" = the majority of the time "all the time" = constantly; at all times	□ Jamais □ Parfois □ Plus de la moitié du temps □ Tout le temps	□ Never □ Sometimes □ More than half the time □ All the time	No revision needed	No revision needed	No revision needed
ILQI ADDITIONAL RESPONSE OPTION FOR ITEM 1 AND 2 □ I am not currently working/studying due to ITP	This is an available response choice "due to" = because of; as a result of "ITP" = immune thrombocytopenia	□ Je ne travaille/n’étudie pas actuellement en raison de ma TPI	□ I am not currently working/studying because of my ITP	No revision needed	No revision needed	No revision needed
ILQI ADDITIONAL RESPONSE OPTION FOR ITEM 1 AND 2 □ I am not currently working/studying due to other reasons (0)	This is an available response choice "due to" = because of; as a result of "other reasons" = reasons other than ITP; other causes	□ Je ne travaille/n’étudie pas actuellement pour d'autres raisons (0)	□ I am not currently working/studying for other reasons (0)	No revision needed	No revision needed	No revision needed
ILQI ITEM 2 How often have you taken time off work or education because of your ITP?	"how often" = how frequently "taken time off work or education" indicates the person was absent from work or school; this could refer to unexpected absences or planned absences that are due to his/her ITP "ITP" = immune thrombocytopenia	À quelle fréquence avez-vous pris des jours de repos au travail ou dans vos études à cause de votre TPI?	How frequently have you taken days off from work or from your studies because of your ITP?	No revision needed	No revision needed	No revision needed
ILQI ITEM 3 How often has your ITP impacted your ability to concentrate on everyday tasks?	"how often" = how frequently "ITP" = immune thrombocytopenia "impacted" = affected "to concentrate" = to focus your attention "everyday tasks" = common tasks; tasks that are typically part of everyday life "task" = piece of work to be done or undertaken; duty; chore	À quelle fréquence votre TPI a-t-elle eu un impact sur votre capacité à vous concentrer sur des tâches de la vie quotidienne ?	How frequently has your ITP had an impact upon your ability to concentrate on the tasks of daily living?	No revision needed	No revision needed	No revision needed
ILQI ITEM 4 How often has your ITP impacted your social life?	"how often" = how frequently "ITP" = immune thrombocytopenia "impacted" = affected "social life" = the domain of an individual's life that concerns engagement or interaction with other people	À quelle fréquence votre TPI a-t-elle eu un impact sur votre vie sociale ?	How frequently has your ITP had an impact upon your social life?	No revision needed	No revision needed	No revision needed
ILQI ITEM 5 How often has your ITP impacted your sex life?	"how often" = how frequently "ITP" = immune thrombocytopenia "impacted" = affected "sex life" = a colloquial term that captures all the aspects of one's life concerning sexual relationships and levels of sexual activity	À quelle fréquence votre TPI a-t-elle eu un impact sur votre vie sexuelle ?	How frequently has your ITP had an impact upon your sex life?	No revision needed	No revision needed	No revision needed
ADDITIONAL RESPONSE OPTION FOR ILQI ITEM 5 □ Not applicable/prefer not to say		□ Ne s’applique pas/Je préfère ne pas répondre	□ Not applicable/I prefer not to answer	No revision needed	No revision needed	No revision needed
ILQI ITEM 6 How often has your ITP impacted your energy levels?	"how often" = how frequently "ITP" = immune thrombocytopenia "impacted" = affected "energy" = the strength and vitality required for sustained physical or mental activity	À quelle fréquence votre TPI a-t-elle eu un impact sur votre niveau d’énergie ?	How frequently has your ITP had an impact upon your energy level?	No revision needed	No revision needed	No revision needed
ILQI ITEM 7 How often has your ITP impacted your undertaking of daily tasks?	"how often" = how frequently "ITP" = immune thrombocytopenia "impacted" = affected "undertaking of" = taking part in; engagement in; management of "daily" = every day; on a daily basis "task" = piece of work to be done or undertaken; duty; chore	À quelle fréquence votre TPI a-t-elle eu un impact sur la réalisation de vos tâches quotidiennes ?	How frequently has your ITP had an impact upon the performance of your daily tasks?	No revision needed	No revision needed	No revision needed
ILQI ITEM 8 How often has your ITP impacted your ability to support people close to you?	"how often" = how frequently "ITP" = immune thrombocytopenia "impacted" = affected "to support people close to you" = to provide emotional support to people that are close to you (close friends, family, etc.)	À quelle fréquence votre TPI a-t-elle eu un impact sur votre capacité à soutenir vos proches ?	How frequently has your ITP had an impact upon your ability to support your loved ones?	No revision needed	Is there an alternative word we can use rather than 'loved ones'? This has the connotation that it is family member, but this item should include supporting anyone close to the patient	Confirmed that in FR "proches" means "close ones" referring to any close family member or friend. The BT is revised for clarity with no revision to the FT needed
ILQI ITEM 9 How often has your ITP negatively impacted your hobbies?	"how often" = how frequently "ITP" = immune thrombocytopenia "impacted" = affected "hobbies" = activities done in one's leisure time for pleasure	À quelle fréquence votre TPI a-t-elle eu un impact négatif sur vos loisirs ?	How frequently has your ITP had a negative impact upon your leisure activities?	No revision needed	No revision needed	No revision needed
ILQI ITEM 10 How often has your ITP negatively impacted your normal capacity to exercise?	"how often" = how frequently "ITP" = immune thrombocytopenia "impacted" = affected "normal capacity to exercise" = typical capacity to engage in physical activity to maintain and/or improve health	À quelle fréquence votre TPI a-t-elle eu un impact négatif sur votre capacité normale à faire du sport ?	How frequently has your ITP had a negative impact upon your normal ability to exercise?	No revision needed	No revision needed	No revision needed
INSTRUCTION AT THE END OF THE ILQI Please check you have answered EVERY question	"check" = make sure; be sure "answered" = responded to "every question" = all questions	Veuillez vérifier que vous avez répondu à TOUTES les questions	Please check that you have answered ALL the questions	No revision needed	No revision needed	No revision needed
END OF THE ILQI Thank you	"thank you" is a closing message to the respondent indicating the end of the questionnaire	Merci	Thank you	Ending punctuation has been added to the FT for correct grammar in the formatted file. The BT has been revised to reflect the updated FT punctuation	No revision needed	No revision needed
SCORING OF ILQI KEY:	The key below provides explanation of each score	LÉGENDE	KEY:	No revision needed	No revision needed	No revision needed
I am not currently working/studying due to ITP = 4	"due to" = because of; as a result of "ITP" = immune thrombocytopenia	Je ne travaille/n’étudie pas actuellement en raison de ma TPI = 4	I am not currently working/studying because of my ITP = 4	No revision needed	No revision needed	No revision needed
Missing:		Manquant:	Missing:	No revision needed	No revision needed	No revision needed
I am not currently working due to other reasons = 0, not applicable/prefer not to say = 0	"due to" = because of; as a result of "other reasons" = reasons other than ITP; other causes	Je ne travaille/n’étudie pas actuellement pour d'autres raisons = 0, Ne s’applique pas/Je préfère ne pas répondre = 0	I am not currently working/studying for other reasons = 0, Not applicable/I prefer not to answer = 0	No revision needed	No revision needed	No revision needed
Min score:	"Min" = minimum	Score min.:	Min. score:	No revision needed	No revision needed	No revision needed
7		7	7	No revision needed	No revision needed	No revision needed
Max score:	"Max" = maximum	Score max.:	Max. score:	No revision needed	No revision needed	No revision needed
40		40	40	No revision needed	No revision needed	No revision needed
Score of 20 or above suggests significantly impaired quality of life	"or above" = or higher "significantly" = sufficiently great or important to be worthy of attention; noteworthy "impaired" = negatively impacted; diminished "quality of life" = the standard of health, comfort, and happiness experienced by an individual or group	Un score égal ou supérieur à 20 suggère une altération significative de la qualité de vie	A score equal to or greater than 20 suggests a significant impairment with regard to life quality	No revision needed	No revision needed	No revision needed
Score of 30 or above suggests severely impaired quality of life	"or above" = or higher "severely" = seriously "impaired" = negatively impacted; diminished "quality of life" = the standard of health, comfort, and happiness experienced by an individual or group	Un score égal ou supérieur à 30 suggère une altération grave de la qualité de vie	A score equal to or greater than 30 suggests a severe impairment with regard to life quality	No revision needed	No revision needed	No revision needed

For most of the sentences/items in the ILQI, the forward and backwards translations into Japanese and French were reconciled with no revision needed. Full details of each step of the translation process for the Japanese version of the ILQI are presented in Table 2. Resolution reasoning was needed for the following Japanese translations:

- For the ILQI instructions, ‘how much’ was back translated as ‘the degree’ as this more accurately reflected the source instrument. Resolution reasoning confirmed that no revision was needed as the translation accurately conveyed the same meaning. In the second sentence of the ILQI instructions, the forward translation of ‘the aim’ had to be revised by removing any reference to ‘another’ and no further revisions were needed.
- For ILQI item 1, the back translation was revised to clarify the meaning of ‘studies’ as being different to ‘schoolwork’ and no further revisions were needed.
- The developer review raised concern that the response options ‘majority of the time’ was not a direct translation of ‘more than half of the time’. Linguistic feedback confirmed that the language was revised to use a more literal translation.
- For IQLI item 2, the back translation of ‘skip work or school’ was revised to ‘take time off work or school’ and no further revisions were needed.
- For ILQI item 3, resolution reasoning identified a difference in the tense between the source instrument and forward translation, which was revised. The concept of ‘work’ was included in the back translation of ‘everyday tasks’ which was removed following the developer review to ensure the item was relevant to those patients who were not working.
- For ILQI item 7, the back translation identified a difference between the use of singular or plural version of ‘task/s’ between the source instrument and the translation, however, resolution reasoning confirmed no revision was needed.
- For ILQI item 8, the forward translation was revised to ensure the term ‘close’ conveyed emotional closeness rather than physical closeness.
- For ILQI item 9, resolution reasoning confirmed that the difference between the use of the singular or plural version of ‘hobby/hobbies’ did not require any revisions.
- For ILQI item 10, the back translation suggested that ‘exercise’ had been translated as ‘motor ability’, which although still represents a similar concept, the wording was revised to more closely reflect the term ‘exercise’, used in the source instrument.
- The translation of the scoring thresholds were revised to reflect the source instrument more closely. For example, following developer review and linguistic feedback, ‘impediments’ was changed to ‘diminishment’ and ‘considerable’ changed to ‘significant.’

Full details relating to each step of the translation process for the French version of the ILQI are presented in Table 3. Resolution reasoning was needed with the following French translations:

- For the ILQI instructions, the back translation of ‘aim’ differed between sentences, with one using the term ‘goal’ and the other using the term ‘objective’. Resolution reasoning confirmed that the translations were both correct based on the context and meaning of the source instrument and no revisions were needed.
- For the second sentence of the ILQI instructions, ‘bleeding’ was back translated as ‘bleeds’. Linguistic feedback confirmed that in French, *‘saignements’* can be translated as ‘bleeds’ or ‘bleeding’ and no revisions were needed.
- For ILQI item 8, the back translation changed ‘support to people that are close to you’ to ‘support your loved ones’. Linguistic feedback confirmed that in French, *‘proches’* can refer to any close family member or friend and no revisions were needed.

### Cognitive interviews with Japanese and French patients

Following the forward and backwards translations, developer review, linguistic feedback and resolution reasoning, the Japanese and French versions of the ILQI were updated. The revised versions were tested in cognitive interviews with five Japanese participants and five French participants, to assess level of understanding and readability of the translated instrument. The demographic characteristics of the participants are presented in Table 4. All interviews were conducted in October and November 2020. The Japanese participants were 40–65 years old, with a mean age of 51 years. Three of the five participants were female and the majority (4/5 participants) had between 14 and 16 years of education. All Japanese participants had been diagnosed with blood clots, with length of diagnosis ranging from 3 to 21 years and a mean diagnosis of 10 years. The French participants were 33–74 years old, with a mean age of 57 years (similar to the Japanese participants). Three of the five participants were female, and all had at least 9 years of education. Four of the five French participants were diagnosed with phlebitis and all participants had been diagnosed with their respective condition for 1 year.

**Table 4:** Demographic characteristics of the participants in the cognitive interviews

Description	Japanese participants	French participants
Age, years, mean (range)	51.4 (40–65)	57 (33–74)
Gender, n		
Female Male	3 2	3 2
Diagnosis, n		
Phlebitis Blood clot disorders Superficial thrombophlebitis	0 5 0	4 0 1
Years since diagnosis, mean (range)	10.2 (3–21)	1 (1–1)
Education level, number of years		
9 12 14–16	0 1 4	3 2 0

The results of the cognitive debriefing interviews for the Japanese ILQI are presented in Table 5 and results of the cognitive debriefing of the French ILQI are presented in Table 6. Most of the instructions and items of the ILQI were well understood by both the Japanese and French participants and no issues were reported.

**Table 5:** Detail of the cognitive debriefing and linguistic feedback for translation of the ILQI into Japanese for Japan

Source (US-English)	Cognitive Debriefing Analysis	Linguist Feedback	Final Forward Translation	Final Back Translation
TITLE OF ILQI ITP Life Quality Index (ILQI)	All respondents understood correctly and had no suggestions	No revision needed	ITP 生活の質指標 (ILQI)	ITP Quality of Life Indicator (ILQI)
FIRST SENTENCE OF ILQI INSTRUCTIONS The aim of this questionnaire is to measure how much your ITP has affected your life OVER THE PAST MONTH	All respondents understood correctly and had no suggestions	No revision needed	この質問票の目的は、過去一ヵ月にわたり、免疫性血小板減少症 (ITP) がどのくらい生活に影響を与えたかを測定することです。	The purpose of this questionnaire is to gauge how much impact immune thrombocytopenia (ITP) has affected your life over the past month
SECOND SENTENCE OF ILQI INSTRUCTIONS The aim is to try to standardise how, besides bleeding, your ITP affects your life	All respondents understood correctly and had no suggestions	No revision needed	目的は、出血以外に、ITPがどのように生活に影響を及ぼすかを標準化することです。	The aim is to standardize how ITP impacts one’s life apart from bleeding
THIRD SENTENCE OF ILQI INSTRUCTIONS Please tick one box	All respondents understood correctly and had no suggestions	No revision needed	1つのボックスに印をつけてください。	Please check one box
ILQI ITEM 1 How often has your ITP impacted on your working life or studies?	All respondents understood correctly and had no suggestions	No revision needed	ITP によって、どのくらいの頻度で仕事や学業に影響がありましたか?	How often did ITP impact your work and/or study?
ILQI RESPONSE OPTIONS □ Never □ Sometimes □ More than half the time □ All the time	All respondents understood correctly and had no suggestions	No revision needed	□ 全くない □ 時々 □ 半分以上の時間 □ 常時	□ Never □ Sometimes □ More than half the time □ Always
ILQI ADDITIONAL RESPONSE OPTION FOR ITEM 1 AND 2 □ I am not currently working/studying due to ITP	All respondents understood correctly and had no suggestions	No revision needed	□ ITP のために現在、仕事や勉強はしていない	□I am not working or studying now because of ITP
ILQI ADDITIONAL RESPONSE OPTION FOR ITEM 1 AND 2 □ I am not currently working/studying due to other reasons (0)	All respondents understood correctly and had no suggestions	No revision needed	□ 他の理由により現在、仕事や勉強はしていない (0)	□I am not working or studying now because of other reasons (0)
ILQI ITEM 2 How often have you taken time off work or education because of your ITP?	All respondents understood correctly and had no suggestions	No revision needed	のために、どのくらいの頻度で仕事を欠勤したり学校を欠席したりしましたか?	How often did you take time off from work or school because of ITP?
ILQI ITEM 3 How often has your ITP impacted your ability to concentrate on everyday tasks?	All respondents understood correctly and had no suggestions	No revision needed	によって、どのくらいの頻度で日常作業を行うための集中力に影響がありましたか?	How often has ITP impacted your concentration to do your daily task?
ILQI ITEM 4 How often has your ITP impacted your social life?	All respondents understood correctly and had no suggestions	No revision needed	ITP によって、どのくらいの頻度で社会生活に影響がありましたか?	How often did ITP impact your social life?
ILQI ITEM 5 How often has your ITP impacted your sex life?	All respondents understood correctly and had no suggestions	No revision needed	によって、どのくらいの頻度で性生活に影響がありましたか?	How often did ITP impact your sex life?
ADDITIONAL RESPONSE OPTION FOR ILQI ITEM 5 □ Not applicable/prefer not to say	All respondents understood correctly and had no suggestions	No revision needed	□全くない□時々□半分以上の時間□常時□該当しない/回答したくない	□ Not applicable/Don’t want to answer
ILQI ITEM 6 How often has your ITP impacted your energy levels?	All respondents understood correctly and had no suggestions	No revision needed	によって、どのくらいの頻度で活力度に影響がありましたか?	How often did ITP impact your level of energy?
ILQI ITEM 7 How often has your ITP impacted your undertaking of daily tasks?	All respondents understood correctly and had no suggestions	No revision needed	によって、どのくらいの頻度で日常作業の遂行に影響がありましたか?	How often did ITP impact the performance of your daily tasks?
ILQI ITEM 8 How often has your ITP impacted your ability to support people close to you?	All respondents understood the item and did not report any difficulties. However, the cognitive interviewer noted that the concepts here indicate that "support" as used in the source, indicates “to provide emotional support”. The interviewer suggested adding “心の[mental]” to the translation and tested the wording on the respondents for understanding. All respondents agreed that this additional wording was clear and that “心の支え[mental support]” is fine if the meaning is “to provide emotional support”	Even though all respondents agreed that the addition of "mental" is appropriate here for referring to "emotional support", during review the client confirmed that they would like this specific term removed from the translation as this should indicate all types of support. As a result, no revision here is needed	によって、どのくらいの頻度で近しい人の支えとなる能力に影響がありましたか?	How often did ITP impact your ability to be support to people close to you?
ILQI ITEM 9 How often has your ITP negatively impacted your hobbies?	All respondents understood correctly and had no suggestions	No revision needed	によって、どのくらいの頻度であなたの趣味に悪影響がありましたか?	How often did ITP negatively impact your hobbies?
ILQI ITEM 10 How often has your ITP negatively impacted your normal capacity to exercise?	All respondents confirmed that they understood correctly and had no suggestions. However, when reviewing their paraphrasing of the item, it was clear that all respondents thought that this item was referring to general movement ability and not exercise specifically. They indicated that this was referring to being able to stand up, walk around, etc	The FT and BT are revised to better align with the source concept of "exercise" rather than daily physical activities (walking in daily life / lifting heavy packages / getting up / taking a shower). The term "ability" has been omitted as it affects natural flow of Japanese but does not impact understanding	によって、どのぐらいの頻度で、スポーツなど健康のための運動に悪影響がありましたか?	How often did ITP negatively impact exercise for your health, such as leisure sports?
INSTRUCTION AT THE END OF THE ILQI Please check you have answered EVERY question	All respondents understood correctly and had no suggestions	No revision needed	すべての質問に回答したことを確認してください。	Please make sure that you have answered all questions
END OF THE ILQI Thank you	All respondents understood correctly and had no suggestions	No revision needed	ありがとうございました。	Thank you

**Table 6:** Detail of the cognitive debriefing and linguistic feedback for translation of the ILQI into French for France

Source (US-English)	Cognitive Debriefing Analysis	Linguist Feedback	Final Forward Translation	Final Back Translation
TITLE OF ILQI ITP Life Quality Index (ILQI)	The word "indice" caused some issues for R1 and R2 but they were able to grasp the general meaning	It is confirmed that this is a valid term and accurately reflects the source use of "Index". No revision needed	Indice de qualité de vie liée à la thrombopénie immune (ITP Life Quality Index, ILQI)	Immune thrombocytopenia-related life quality index (ITP Life Quality Index, ILQI)
FIRST SENTENCE OF ILQI INSTRUCTIONS The aim of this questionnaire is to measure how much your ITP has affected your life OVER THE PAST MONTH	All respondents understood this item and no issues were reported	No revision needed	L’objectif de ce questionnaire est de mesurer l’impact que la thrombopénie immune (TPI) a eu sur votre vie AU COURS DU DERNIER MOIS	The objective of this questionnaire is to measure the impact that immune thrombocytopenia (ITP) has had upon your life OVER THE PAST MONTH
SECOND SENTENCE OF ILQI INSTRUCTIONS The aim is to try to standardise how, besides bleeding, your ITP affects your life	R1, R2, R3 and R4 stated having problems understanding the term "standardiser". R2, R3 and R5's rewording ("they want to have an overall idea of how ITP affects life") clearly shows the intended meaning was not understood properly	The misunderstanding by the majority of respondents is most likely due to the fact that "standardiser" in French means "make sure something comes in conformity with a set standard" (i.e. in this context, make all persons experience the disease in the same way). It cannot mean "regulate" as in "avoid ups and downs" for one given person. The FT and BT have been revised to clarify the source meaning of "standardise" in the FT	Le but est d'essayer de réguler l'impact de la TPI sur votre vie, en dehors des saignements	The goal is to try to regulate the impact of ITP on your life, apart from bleeding
THIRD SENTENCE OF ILQI INSTRUCTIONS Please tick one box	All respondents understood this item and no issues were reported	No revision needed	Veuillez cocher une case	Please check off one box
ILQI ITEM 1 How often has your ITP impacted on your working life or studies?	All respondents understood this item and no issues were reported	No revision needed	À quelle fréquence votre TPI a-t-elle eu un impact sur votre vie professionnelle ou vos études ?	How frequently has your ITP had an impact upon your professional life or your studies?
ILQI RESPONSE OPTIONS □ Never □ Sometimes □ More than half the time □ All the time	All respondents understood this item and no issues were reported	No revision needed	□Jamais □ Parfois □ Plus de la moitié du temps □ Tout le temps	□ Never □ Sometimes □ More than half the time □ All the time
ILQI ADDITIONAL RESPONSE OPTION FOR ITEM 1 AND 2 □ I am not currently working/studying due to ITP	All respondents understood this item and no issues were reported	No revision needed	□Je ne travaille/n’étudie pas actuellement en raison de ma TPI	□ I am not currently working/studying because of my ITP
ILQI ADDITIONAL RESPONSE OPTION FOR ITEM 1 AND 2 □ I am not currently working/studying due to other reasons (0)	All respondents understood this item and no issues were reported	No revision needed	□ Je ne travaille/n’étudie pas actuellement pour d'autres raisons (0)	□ I am not currently working/studying for other reasons (0)
ILQI ITEM 2 How often have you taken time off work or education because of your ITP?	R1, R3 and R4 pointed out that "jours de repos" sounds as if it is a personal choice, or even a planned non-working day. All three respondents suggested to use "arrêt de travail" (sick leave) instead	The suggestion made by all three respondents is not ideal as it would imply leave from work that was prescribed by a doctor. The notion of "absence" would be more appropriate and less restrictive so as to incorporate any time away due to illness. The FT and BT are revised	À quelle fréquence avez-vous été absent(e) au travail ou de vos études à cause de votre TPI ?	How frequently have you been absent from work or from your studies because of your ITP?
ILQI ITEM 3 How often has your ITP impacted your ability to concentrate on everyday tasks?	All respondents understood this item and no issues were reported	No revision needed	À quelle fréquence votre TPI a-t-elle eu un impact sur votre capacité à vous concentrer sur des tâches de la vie quotidienne ?	How frequently has your ITP had an impact upon your ability to concentrate on the tasks of daily living?
ILQI ITEM 4 How often has your ITP impacted your social life?	All respondents understood this item and no issues were reported	No revision needed	À quelle fréquence votre TPI a-t-elle eu un impact sur votre vie sociale ?	How frequently has your ITP had an impact upon your social life?
ILQI ITEM 5 How often has your ITP impacted your sex life?	All respondents understood this item and no issues were reported	No revision needed	À quelle fréquence votre TPI a-t-elle eu un impact sur votre vie sexuelle ?	How frequently has your ITP had an impact upon your sex life?
ADDITIONAL RESPONSE OPTION FOR ILQI ITEM 5 □ Not applicable/prefer not to say[/g5]	All respondents understood this item and no issues were reported	No revision needed	□ Ne s’applique pas/Je préfère ne pas répondre	□ Not applicable/I prefer not to answer
ILQI ITEM 6 How often has your ITP impacted your energy levels?	All respondents understood this item and no issues were reported	No revision needed	À quelle fréquence votre TPI a-t-elle eu un impact sur votre niveau d’énergie ?	How frequently has your ITP had an impact upon your energy level?
ILQI ITEM 7 How often has your ITP impacted your undertaking of daily tasks?	All respondents understood this item and no issues were reported	No revision needed	À quelle fréquence votre TPI a-t-elle eu un impact sur la réalisation de vos tâches quotidiennes ?	How frequently has your ITP had an impact upon the performance of your daily tasks?
ILQI ITEM 8 How often has your ITP impacted your ability to support people close to you?	All respondents understood this item and no issues were reported	No revision needed	À quelle fréquence votre TPI a-t-elle eu un impact sur votre capacité à soutenir vos proches ?	How frequently has your ITP had an impact upon your ability to support your close ones?
ILQI ITEM 9 How often has your ITP negatively impacted your hobbies?	All respondents understood this item and no issues were reported	No revision needed	À quelle fréquence votre TPI a-t-elle eu un impact négatif sur vos loisirs ?	How frequently has your ITP had a negative impact upon your leisure activities?
ILQI ITEM 10 How often has your ITP negatively impacted your normal capacity to exercise?	R1, R3 and R4 pointed out that "normal" sounds awkward, and R2 said it sounded "judgmental". In French, "normal" has a "normative" connotation. They all suggested "usual" instead	The suggested change by the respondents to use "usual" would be a better option as it would refer to the usual capacity of a person to exercise, and would avoid the idea of a judgement as to what is normal and what is not. The FT and BT are revised Although not mentioned by any respondents during the interviews, it should be noted that "faire du sport" in French does not include low intensity exercise like walking, for example. Since many people, depending on the severity of the disease, may be able to go out for a walk but may be totally unable to "play sports" (i.e. a certain degree of intensity) the FT is also revised to use a more literal translation for "exercise" which could include any intensity level. No revision to the BT for this latter change	À quelle fréquence votre TPI a-t-elle eu un impact négatif sur votre capacité habituelle à faire de l'exercice ?	How frequently has your ITP had a negative impact upon your usual ability to exercise?
INSTRUCTION AT THE END OF THE ILQI Please check you have answered EVERY question	All respondents understood this item and no issues were reported	No revision needed	Veuillez vérifier que vous avez répondu à TOUTES les questions	Please check that you have answered ALL the questions
END OF THE ILQI Thank you	All respondents understood this item and no issues were reported	No revision needed	Merci!	Thank you!

Linguistic feedback and input was required for the following Japanese translations:

- While all Japanese participants appeared to understand ILQI item 10, review of their paraphrasing suggested that all participants thought this item was referring to general movement ability rather than exercise. The item wording was revised to better align with the source instrument and relevance confirmed with forward and backwards translations.

Linguistic feedback and input was required for the following French translations:

- Despite two participants reporting some problems with the term *‘indice’* in the title of the ILQI, linguistic feedback confirmed that this term accurately reflects the source instrument and the participants were able to understand the overall meaning of the title.
- 4/5 French participants misunderstood the translation of ‘standardise’, therefore, the forward and backwards translations were revised to clarify this misunderstanding.
- 3/5 French participants reported that the term *‘jours de repos’*, in ILQI item 2, was not a true reflection of ‘take time off work’. Item wording was revised, and final forward and backwards translations reflect the source instrument.
- 3/5 French participants reported that the term ‘normal’, used in ILQI item 10 can sound judgemental in French and suggested replacing this with a translation of ‘usual’. The item wording was revised, and translation confirmed with final forward and backwards translation.

### Review by ITP experts

The final step in the linguistic validation process was the review of the translated and cognitively debriefed ILQI by experts in the field of ITP, from Japan and France. While the French ITP expert did not suggest any fundamental changes to the ILQI, some modifications were made to simplify the questions for the patients. Similarly, the Japanese ITP expert did not make any changes to the content of the ILQI but rather modified and softened some of the language to make the Japanese version more culturally appropriate for Japanese patients. The final versions were proof-read by the linguistic experts and proof-reading certificates were issued to confirm that the changes made by the French and Japanese experts did not change the translations and linguistic validation work conducted to date.

## Discussion

As the clinical assessments of ITP often focus on platelet counts and risk of bleeding, there was an unmet need for a valid and reliable PRO assessment to focus on the under-reported HRQoL impacts associated with ITP. The ILQI was developed to address this unmet need and the US-English version has been rigorously developed according to regulatory guidance and has demonstrated good content validity and psychometric properties. To ensure the ILQI could be administered as a tool for clinical practice in Japan and France, it was necessary for the ILQI to be translated and undergo the linguistic validation process.

Findings from the DIF, conducted as part of the psychometric analyses, identified differences in the way patients from the USA and patients from non-Western countries (including Japan) answered most items on the ILQI. This highlighted that further translational work and linguistic validation work was needed to ensure the ILQI was appropriate and culturally relevant to be used as a tool for clinical practice in Japan. Additionally, while the DIF confirmed that patients with ITP in France answered most items in a similar way to the patients with ITP in the USA, to have confidence that the ILQI is suitable for use in clinical practice in France, it was also necessary to conduct full linguistic validation analysis on the French version of the ILQI. The ILQI was translated and linguistically validated in accordance with the best practice guidelines, according to the ISPOR Task Force.

**Fig. 2:**
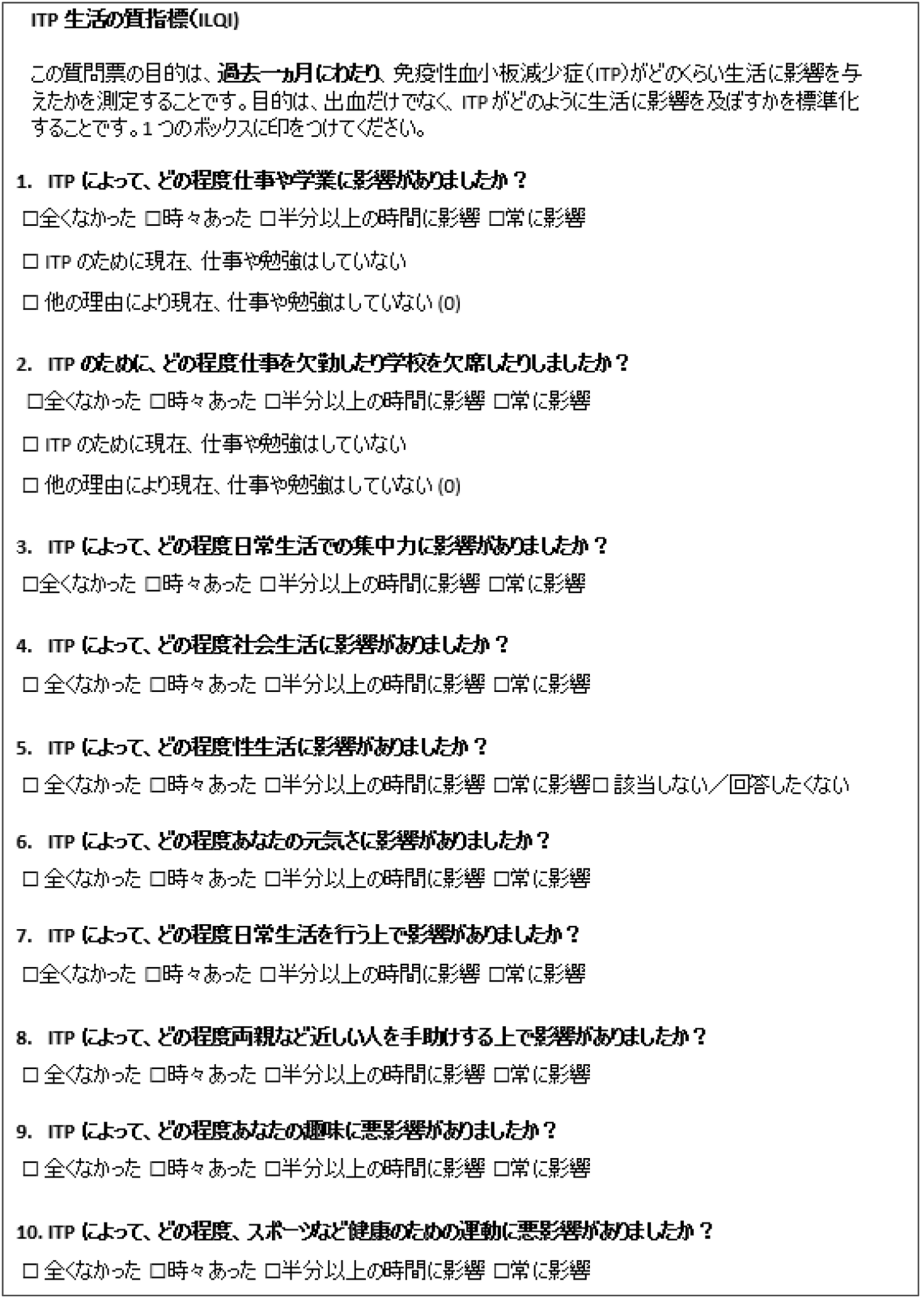
ILQI Japanese version following translation and linguistic validation

**Fig. 3:**
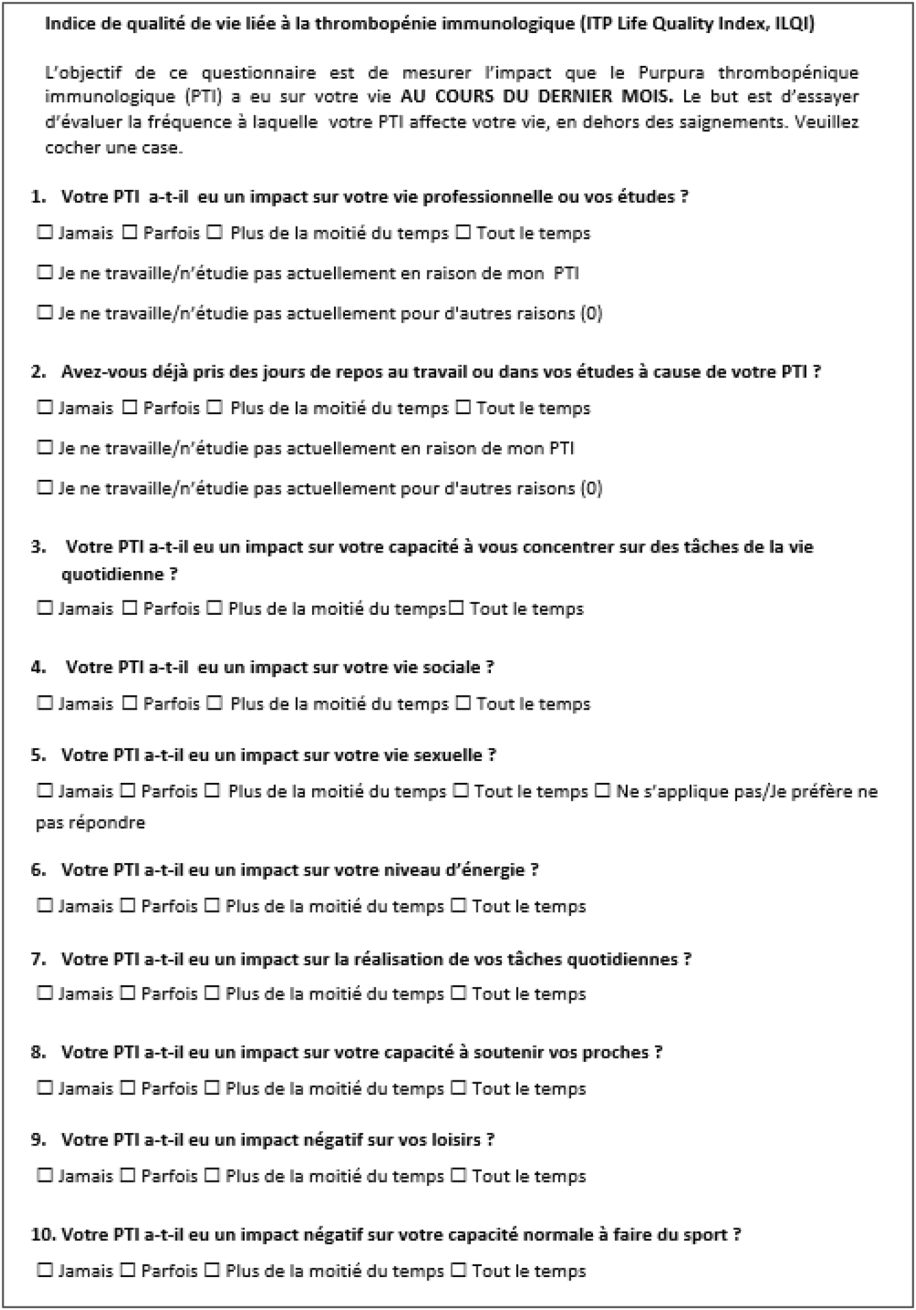
ILQI French version following translation and linguistic validation

As expected, the linguistic validation process identified words or phrases that were not interpreted as intended and subtle changes were made to both the French and Japanese versions of the ILQI to improve understanding and cultural relevance. The final versions of the translated and linguistically validated Japanese ILQI is presented in Fig. 2 and final version of the French ILQI is presented in Fig. 3.

While the validation was conducted in accordance with best practice guidelines, there are some limitations of this study which need to be considered. The cognitive interviews were conducted with only five patients from each county; while, a larger sample size may have provided more evidence to support the understanding of the items and instructions, it is noted that according to the COSMIN group, a sample size of 4–6 is considered to be adequate, a sample size of ≥ 7 is considered to be very good [14]. Also, while all patients were diagnosed with a hematological condition, none of the patients were diagnosed specifically with ITP and, therefore, conducting more cognitive interviews with patients diagnosed with ITP would provide further support for the cultural validity of the ILQI. Future work should focus on conducting linguistic validation analysis in other countries, to ensure the ILQI is appropriate for use in clinical practice in other countries, in addition to the UK/USA, Japan and France.

In conclusion, the ILQI is ready and available for use in clinical practice in the UK/USA, Japan and France and content validity, psychometric validity and linguistic validity have been confirmed. Implementing the ILQI into clinical practice across different countries should help to aid discussions between patients and clinicians, inform treatment decisions and improve the overall HRQoL, comprehensively reflecting experiences of demographically diverse patients with ITP.
